# Distributed Group Location Update Algorithm for Massive Machine Type Communication

**DOI:** 10.3390/s20247336

**Published:** 2020-12-21

**Authors:** Mincheol Paik, Haneul Ko

**Affiliations:** Department of Computer and Information Science, Korea University, Sejong 30019, Korea; fall123123@korea.ac.kr

**Keywords:** massive machine type communication (mMTC), Internet of Things (IoT), distributed group location update algorithm, location update, nash equilibrium

## Abstract

Frequent location updates of individual Internet of Things (IoT) devices can cause several problems (e.g., signaling overhead in networks and energy depletion of IoT devices) in massive machine type communication (mMTC) systems. To alleviate these problems, we design a distributed group location update algorithm (DGLU) in which geographically proximate IoT devices determine whether to conduct the location update in a distributed manner. To maximize the accuracy of the locations of IoT devices while maintaining a sufficiently small energy outage probability, we formulate a constrained stochastic game model. We then introduce a best response dynamics-based algorithm to obtain a multi-policy constrained Nash equilibrium. From the evaluation results, it is demonstrated that DGLU can achieve an accuracy of location information that is comparable with that of the individual location update scheme, with a sufficiently small energy outage probability.

## 1. Introduction

Recently, the Internet of Things (IoT) has been rapidly adopted all over the world [1,2,3]. Because of this explosive popularity, massive machine type communication (mMTC) has been defined as one of main communication types in 5G networks [4,5,6]. One of the primary application scenarios of mMTC is intelligent transportation systems (ITSs) [7,8,9,10], where many IoT devices (e.g., GPS devices and various types of sensors) are deployed in vehicles. With the assistance of IoT devices, various useful services can be provided to users, especially when the locations of IoT devices are well managed [11]. For example, consider a package tracking system in which the tracking IoT devices are attached to each package in the vehicle, and these IoT devices update their locations to the location management server. The package tracking service can then be easily achieved. In addition, we can consider a case where people have more than 2∼3 wearable devices (e.g., smart watch, health monitoring devices, etc.) in their bodies and these devices update their location for advanced services (e.g., location-based advertisement, location tracking, etc.). However, when all the IoT devices update their locations individually, a significant signaling overhead can occur in networks. In addition, because the signaling process and message transmission for location updates generally consume a large amount of energy, frequent location updates can cause energy depletion of IoT devices. Many works have addressed this problem [12,13,14,15,16,17,18,19,20,21,22,23]. One of the intuitive solutions is electing a representative IoT device (i.e., group leader). Then, only a group leader updates its location (which is probably the same as locations of the other IoT devices). Even though this approach can reduce unnecessary location updates, the procedure of electing a group leader needs considerable signalings. In addition, when the group leader fails, the whole system can be disrupted.

In this paper, we propose a distributed group location update algorithm (DGLU) for massive machine type communication (mMTC). In DGLU, when geographically proximate IoT devices move to another location, they determine whether to conduct the location update in a distributed manner. Because each IoT device cannot know the other IoT devices’ decision, some degree of redundant location updating may occur. In other words, to achieve a sufficient accuracy of location information, some redundant location updates are unavoidable. However, excessively redundant location updates decrease the energy efficiency of IoT devices. To optimize this trade-off, we formulate a constrained stochastic game model that can be converted into an equivalent linear programming (LP). We then design a best response dynamics-based algorithm to reach a multi-policy constrained Nash equilibrium. The evaluation results show that DGLU can achieve an accuracy of location information that is comparable with the individual location update scheme while controlling the energy outage probability below the desired level. Moreover, it is observed that DGLU can be adopted in practical systems due to the fast convergence property of the proposed iteration algorithm.

We summarize the contributions of this paper as follows: (1) to the best our knowledge, this is the first work to optimize the trade-off between the accuracy of location information and the energy efficiency of IoT devices in a distributed manner (i.e., based on a constrained stochastic game); (2) the optimal location update policy can be obtained with a few iterations, which indicates that the policy can be adopted by practical systems without an excessive overhead; and (3) the presented evaluation results under various environments can provide invaluable guidelines for constructing mMTC systems.

The remainder of this paper is organized as follows. Related works are summarized in Section 2 and DGLU is elaborated in Section 3. Then, the constrained stochastic game model is formulated in Section 4. Evaluation results are presented in Section 5, and followed by the conclusion in Section 6.

## 2. Related Work

Several studies were conducted on mobility management in IoT systems [12,13,14,15,16,17,18,19,20,21,22,23,24,25,26,27]. These can be categorized into: (1) mobility management for an individual device [12,13,14,15,16]; (2) mobility management for a group of devices [17,18,19,20,21,22,23]; and (3) mobility management for the privacy of devices [24,25,26,27].

Fu et al. [12] proposed a delayed location update algorithm where mobile devices postpone their location updates until the the timer expiration to decrease the number of updates. Pacheco-Paramo et al. [13] designed two location management schemes with the objective reducing signaling overhead with a sufficient accuracy of location information of mobile devices. Ko et al. [14] proposed an adaptive location update algorithm, where mobile devices update their location only at specific locations where they remain for a long time. Yu and Gu [15] introduced a cost-efficient location management scheme where a mobile device conducts location updates only in chosen locations by considering its current status. Wang et al. [16] proposed an Q-learning-based mobility management scheme where a mobile device makes a handover decision by observing the task delay. However, the feature of group mobility was not investigated in these studies [12,13,14,15,16].

Wang et al. [17] proposed a group location update method in which two leader selection methods, regular and irregular selection methods, are used depending on whether a previous group leader exists or not. Fu et al. [18] suggested a group-based mobility management scheme where the location management server divides mobile devices into several groups by considering the similarity of their mobility patterns, and a leader device conducts location update on behalf of the other devices in the same group. Li and Wu [19] introduced a delay-based location management scheme for a group of mobile devices, in which each mobile device updates its location after a random time delay to reduce simultaneous location update. Suzuki et al. [20] developed a low-complexity mobility detection mechanism based on signaling data of mobile devices. Chowdhury et al. [21] proposed a resource management method for group mobility that includes a bandwidth adaptation policy and a dynamic bandwidth reservation policy. Dooren et al. [22] proposed a group handover scheme to allow for the simultaneous handover of a group of mobile devices and evaluated its performance based on derived analytical models for handover delay. Chatzikokolakis et al. [23] proposed a location management scheme where only one device performs location update on behalf of a group formed for the application specific purpose.

Aman et al. [24] proposed a location-based authentication protocol that exploits physical unclonable functions to establish a root of trust, secure key generation, and hardware authentication. Albouq et al. [25] introduced a privacy protection approach by integrating obfuscation and trust third party approaches and exploited cahching and mix-zone technologies to improve its efficiency. Saia et al. [26] introduced a blockchain-based distributed paradigm to exchange data between IoT devices in a private manner. Sun et al. [27] analyzed an existing dummy location selection algorithm protecting the location privacy of IoT devices, an then proposed a new dummy location privacy algorithm that optimizes a tradeoff between the computational cost and the privacy requirement of IoT device.

However, no previous work optimized the location update strategy of multiple devices in a distributed manner based on a constrained stochastic game.

## 3. Distributed Group Location Update Algorithm (Dglu)

**Figure 1:**
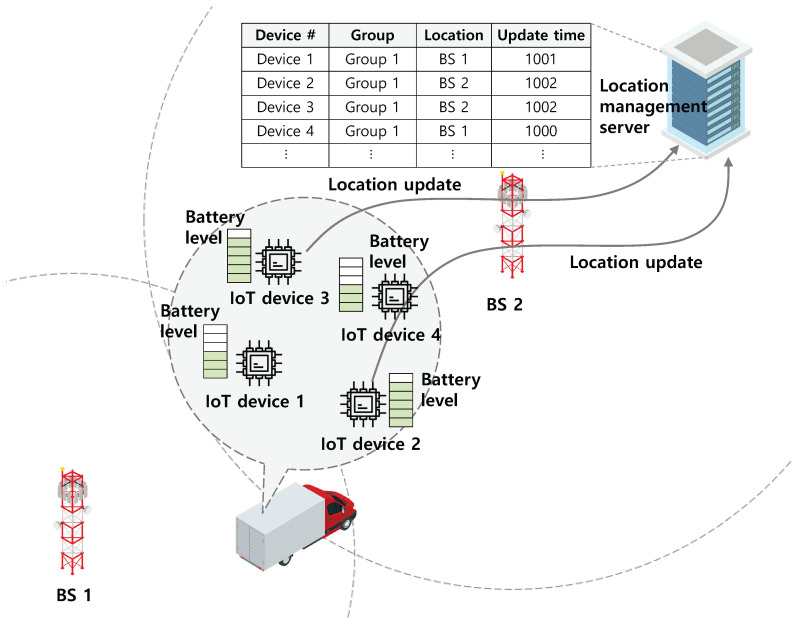
System model.

The system model in this paper is shown in Figure 1. In our system model, it is assumed that several IoT devices are attached to a primary object (e.g., person and vehicles), and these IoT devices have energy harvesting capability (i.e., IoT devices can charge their batteries whose capacity is denoted by $E_{\max}$). Meanwhile, due to the mobility of the primary object, the locations of the IoT devices change, and therefore these devices update their locations to the location management server. However, when all IoT devices update their locations individually, a significant signaling overhead can occur in networks. In addition, because the signaling process and message transmission for location updates generally consume a large amount of energy, frequent location updates can deplete the energy of IoT devices even though they have harvesting capability. Therefore, we introduce a concept of group mobility management, i.e., due to the proximity of IoT devices, the locations of all devices in one group can be managed when only the representative IoT device (i.e., the group leader) updates its location. A group leader can be elected in a distributed manner or centralized manner, but the election procedure leads to a significant signaling overhead. In addition, when the group leader is out-of-order, the whole system cannot operate well. To mitigate these problems, in DGLU, when the locations of geographically proximate IoT devices change, they determine whether to conduct the location update in a distributed manner. To avoid degradation of the accuracy of location information, some redundant location updates need to be allowed. However, when too many IoT devices conduct location updates redundantly, their energy efficiency can decrease. Thus, each IoT device should consider neighboring IoT devices’ updates in the same group and determine whether to conduct location update. For the optimal decision on location update, a constrained stochastic game model is formulated in Section 4. If an IoT device decides to update its location, it transmits a location update message including some information such as its identification, group identification, location, and update time. After receiving the location update message from IoT devices, the location management server updates its database based on the information included in the message.

Meanwhile, when a corresponding device (e.g., a package tracking service provider) wants to find the location of a specific IoT device, it can request the location management server for its location. After receiving this request, the location management server first checks the group of the IoT device. It then provides the location of any IoT device with the latest update time in that group. For example, in Figure 1, because IoT devices 2 and 3 have the latest update time, the location management service provides the location of IoT device 2 or 3 to the requester.

## 4. Constrained Stochastic Game

For a distributed optimal decision on location update to maximize the location accuracy while maintaining the energy outage probability below a desired level, a constrained stochastic game model is formulated [28]. In our game model, *N* IoT devices are the players, and IoT device *i* and player *i* are used interchangeably. Important notations for the constrained stochastic game model are summarized in Table 1.

### 4.1. Local State Space

Each player *i* (i.e., IoT device *i*) has a finite local state space $S_{i}$. A global state space *S* can then be denoted by $\prod_{i}S_{i}$, where ∏ is the Cartesian product. The local state space of player *i* can be defined as

(1) $$S_{i}=L_{i}\times U_{i}\times E_{i}$$

where $L_{i}$ and $U_{i}$ represent the state spaces denoting the current location of IoT device *i* and the registered location of IoT device *i* at the location management server, respectively. Also, $E_{i}$ means the state space representing the energy of IoT device *i*.

When the number of locations in the systems is $L_{\max}$, $L_{i}$ and $U_{i}$ can be described as

(2) $$L_{i}=\left\{1,2,3,\ldots ,L_{\max}\right\}$$

and

(3) $$U_{i}=\left\{1,2,3,\ldots ,L_{\max}\right\},$$

respectively.

Meanwhile, $E_{i}$ can be represented by

(4) $$E_{i}=\left\{0,1,2,\ldots ,E_{\max}\right\}.$$

### 4.2. Local Action Space

Each player *i* has a finite local action space $A_{i}$. Then, a global action space *A* can be defined as $\prod_{i}A_{i}$. Since each IoT device *i* has two options (i.e., conduct location update and does not conduct location update), $A_{i}$ can be defined by

(5) $$A_{i}=\left\{0,1\right\}$$

where $A_{i}=0$ means that IoT device *i* does not update its location, whereas $A_{i}=1$ indicates that IoT device *i* conducts location update.

### 4.3. Transition Probability

The location of IoT device *i* at the location management server is updated based on the current location of IoT device *i* when it conducts a location update, i.e., the transition of $U_{i}$ is affected by $L_{i}$ and $A_{i}$. In addition, when conducting location update, IoT device *i* consumes energy. The transitions of $E_{i}$ are influenced by $A_{i}$. Meanwhile, all states (i.e., $L_{i}$, $U_{i}$, and $E_{i}$) transit independently from each other. Therefore, for an chosen action $A_{i}$, the transition probability $P[S_{i}^{\prime }|S_{i},A_{i}]$ from the current state $S_{i}=\left[L_{i},U_{i},E_{i}\right]$ to the next state $S_{i}^{\prime }=\left[L_{i}^{\prime },U_{i}^{\prime },E_{i}^{\prime }\right]$ can be described as

(6) $$P\left[S_{i}^{\prime }|S_{i},A_{i}\right]=P\left[L_{i}^{\prime }|L_{i}\right]\times P\left[U_{i}^{\prime }|U_{i},L_{i},A_{i}\right]\times P\left[E_{i}^{\prime }|E_{i},A_{i}\right].$$

We assume that the probability of occurrence of a location change for IoT device *i* is $\rho_{L_{i}}$, which is inversely proportional to the residence time in $L_{i}$. Therefore, $P\left[L_{i}^{\prime }|L_{i}\right]$ can be derived as

(7) $$P\left[L_{i}^{\prime }|L_{i}\right]=\left\{\begin{array}{cc} \rho_{L_{i}}P_{L_{i}L_{i}^{\prime }}, & \mathrm{if}\;L_{i}^{\prime }\neq L_{i}\\ 1-\rho_{L_{i}}, & \mathrm{if}\;L_{i}^{\prime }=L_{i}\\ 0, & \mathrm{otherwise} \end{array}\right.$$

where $P_{L_{i}L_{i}^{\prime }}$ is the probability that IoT device *i* moves from $L_{i}$ to $L_{i}^{\prime }$.

When IoT device *i* does not update its location (i.e., $A_{i}=0$), its location registered at the location management server does not change (i.e., $U_{i}^{\prime }=U_{i}$). On the other hand, when IoT device *i* conducts location update (i.e., $A_{i}=1$), its location registered at the location management server is updated based on the current location of IoT device *i* (i.e., $U_{i}^{\prime }=L_{i}$). Therefore, the corresponding transition probabilities can be represented by

(8) $$P\left[U_{i}^{\prime }|U_{i},L_{i},A_{i}=0\right]=\left\{\begin{array}{c} 1,\;\mathrm{if}\;U_{i}^{\prime }=U_{i}\\ 0,\;\mathrm{otherwise} \end{array}\right.$$

and

(9) $$P\left[U_{i}^{\prime }|U_{i},L_{i},A_{i}=1\right]=\left\{\begin{array}{c} 1,\;\mathrm{if}\;U_{i}^{\prime }=L_{i}\\ 0,\;\mathrm{otherwise}. \end{array}\right.$$

IoT devices can harvest energy only if they are in an appropriate situation (e.g., solar-based harvesting IoT device should be located at a sunny spot for the harvesting). Thus, a Bernoulli random process taking values in {0,1} can be exploited to model whether IoT devices harvest one unit of energy or not, i.e., it is assumed that IoT devices harvest (or do not harvest) one unit of energy with the probability $P_{H}$ (or $1-P_{H}$) [29]. Therefore, in our system model, when IoT device *i* with non-fully charged battery (i.e., $E_{i}\neq E_{\max}$) does not update its location to the location management server, its energy $E_{i}$ increases by one unit with probability $P_{H}$. If the battery of IoT device *i* is fully charged (i.e., $S_{i}=E_{\max}$), the energy of IoT device *i* does not increase, because it cannot harvest energy anymore. To sum up, the corresponding probabilities can be described by

(10) $$P\left[E_{i}^{\prime }|E_{i}\neq E_{\max},A_{i}=0\right]=\left\{\begin{array}{cc} P_{H}, & \mathrm{if}\;E_{i}^{\prime }=E_{i}+1\\ 1-P_{H}, & \mathrm{if}\;E_{i}^{\prime }=E_{i}\\ 0, & \mathrm{otherwise} \end{array}\right.$$

and

(11) $$P\left[E_{i}^{\prime }|E_{i}=E_{\max},A_{i}=0\right]=\left\{\begin{array}{c} 1,\;\mathrm{if}\;E_{i}^{\prime }=E_{i}\\ 0,\;\mathrm{otherwise}. \end{array}\right.$$

IoT devices can harvest energy only if they are in an appropriate situation (e.g., solar-based harvesting IoT device should be located at a sunny spot for the harvesting). Thus, a Bernoulli random process taking values in {0,1} can be exploited to model whether IoT devices harvest one unit of energy or not, i.e., it is assumed that IoT devices harvest (or do not harvest) one unit of energy with the probability $P_{H}$ (or $1-P_{H}$) [29]. Therefore, in our system model, when IoT device *i* with a non-fully charged battery (i.e., $E_{i}\neq E_{\max}$) does not update its location to the location management server, its energy $E_{i}$ increases by one unit with probability $P_{H}$. If the battery of IoT device *i* is fully charged (i.e., $S_{i}=E_{\max}$), the energy of IoT device *i* does not increase, because it cannot harvest energy anymore. To sum up, the corresponding probabilities can be described by

(12) $$P\left[E_{i}^{\prime }|E_{i}\neq E_{\max},A_{i}=0\right]=\left\{\begin{array}{cc} P_{H}, & \mathrm{if}\;E_{i}^{\prime }=E_{i}+1\\ 1-P_{H}, & \mathrm{if}\;E_{i}^{\prime }=E_{i}\\ 0, & \mathrm{otherwise} \end{array}\right.$$

and

(13) $$P\left[E_{i}^{\prime }|E_{i}=E_{\max},A_{i}=0\right]=\left\{\begin{array}{c} 1,\;\mathrm{if}\;E_{i}^{\prime }=E_{i}\\ 0,\;\mathrm{otherwise}. \end{array}\right.$$

When IoT device *i* conducts location update (i.e., $A_{i}=1$), it consumes one unit of energy. However, if IoT device *i* does not have any energy (i.e., $E_{i}=0$), it cannot conduct location update. In this situation, IoT device *i* does not consume any energy. Also, its energy can increase by one unit with probability $P_{H}$. Therefore, $P[E_{i}^{\prime }|E_{i}\neq 0,A_{i}=1]$ and $P[E_{i}^{\prime }|E_{i}=0,A_{i}=1]$ can be represented as

(14) $$P\left[E_{i}^{\prime }|E_{i}\neq 0,A_{i}=1\right]=\left\{\begin{array}{cc} P_{H}, & \mathrm{if}\;E_{i}^{\prime }=E_{i}\\ 1-P_{H}, & \mathrm{if}\;E_{i}^{\prime }=E_{i}-1\\ 0, & \mathrm{otherwise} \end{array}\right.$$

and

(15) $$P\left[E_{i}^{\prime }|E_{i}=0,A_{i}=1\right]=\left\{\begin{array}{cc} P_{H}, & \mathrm{if}\;E_{i}^{\prime }=E_{i}+1\\ 1-P_{H}, & \mathrm{if}\;E_{i}^{\prime }=E_{i}\\ 0, & \mathrm{otherwise}. \end{array}\right.$$

### 4.4. Reward Function

To maximize the accuracy of location of IoT device *i*, we define a reward function. When the current location of IoT device *i* is the same as the registered location at the location management server (i.e., $L_{i}=U_{i}$), a corresponding device can obtain the accurate location of IoT device *i*. Even though the current location of IoT device *i* is not same as the registered location at the location management server (i.e., $L_{i}\neq U_{i}$), if at least one IoT device except IoT device *i* has conducted location update, the location management server can provide the accurate location of IoT device *i* to a corresponding device. Therefore, the reward function can be defined as

(16) $$r\left(S_{i},A_{i}\right)=\left\{\begin{array}{cc} 1, & \mathrm{if}\;L_{i}=U_{i}\\ \lambda_{-i}, & \mathrm{if}\;L_{i}\neq U_{i} \end{array}\right.$$

where $\lambda_{-i}$ represents the probability that at least one IoT device except IoT device *i* has updated its location to the location management server.

### 4.5. Constraint Function

To prevent the situation where the energy outage probability becomes higher than a certain level, the constraint function $c\left(S_{i},A_{i}\right)$ is defined. An energy outage implies that the current energy of IoT device *i* is 0. Thus, the constraint function $c\left(S_{i},A_{i}\right)$ can be defined as

(17) $$c\left(S_{i},A_{i}\right)=\left\{\begin{array}{c} 1,\;\mathrm{if}\;E_{i}=0\\ 0,\;\mathrm{otherwise}. \end{array}\right.$$

### 4.6. Optimization Formulation

Let $\pi_{i}$ and π be a stationary policy of IoT device *i* and a stationary multi-policy of all IoT devices, respectively. Then, the long-term average probability that the accurate location of IoT device *i* can be provided to a corresponding device can be defined as

(18) $$\zeta_{E}\left(\pi \right)=\lim_{T\to \infty }\frac{1}{T}\sum_{t=1}^{T}E_{\pi }\left[r\left(S^{t},A^{t}\right)\right]$$

where $S^{t}$ and $A^{t}$ are the global state and the action at time *t*, respectively.

The one of objective of IoT device *i* (i.e., constraint) is to maintain a long-term average energy outage probability below a certain level, which can be described by

(19) $$\xi_{E}\left(\pi \right)=\lim_{T\to \infty }\frac{1}{T}\sum_{t=1}^{T}E_{\pi }\left[c\left(S^{t},A^{t}\right)\right]\leq \theta_{E}$$

where $\theta_{E}$ means the target average energy outage probability.

The multi-policy $\pi^{*}=\left(\pi_{i}^{*},\pi_{-i}^{*}\right)$ is the constrained Nash equilibrium, which is the solution of the formulated constrained stochastic game, if $\zeta_{E}\left(\left(\pi_{i}^{*},\pi_{-i}^{*}\right)\right)\geq \zeta_{E}\left(\left(\pi_{i},\pi_{-i}^{*}\right)\right)$ for each IoT device *i* among any $\pi_{i}$ such that $\left(\pi_{i},\pi_{-i}^{*}\right)$ is feasible (i.e., the constraint is satisfied). In the constrained Nash equilibrium, IoT device *i* cannot achieve a higher location accuracy with any other stationary policy $\pi_{i}$ while the other IoT devices do not change their stationary policies or IoT device *i* will violate the constraint.

For the multi-policy constrained Nash equilibrium, the best response policy $\pi_{i}^{*}$ of IoT device *i* given any stationary policies of other IoT devices $\pi_{-i}$ can be defined as

(20) $$\zeta_{E}\left(\left(\pi_{i}^{*},\pi_{-i}\right)\right)\leq \zeta_{E}\left(\left(\pi_{i},\pi_{-i}\right)\right).$$

LP can then be exploited to obtain the best response policy of IoT device *i*. Let $\phi_{i,\pi_{-i}}\left(S_{i},A_{i}\right)$ be the stationary probability in local state $S_{i}$ and action $A_{i}$ of IoT device *i* given the stationary policies of other IoT devices $\pi_{-i}$. Then, the solution of the equivalent LP model $\phi_{i,\pi_{-i}}^{*}\left(S_{i},A_{i}\right)$ can be mapped to the best response policy of the constrained stochastic game [30,31]. The equivalent LP model can be defined by

(21) $$\max_{\phi (S,A)}\sum_{S}\sum_{A}\phi_{i,\pi_{-i}}\left(S_{i},A_{i}\right)r\left(S_{i},A_{i}\right)$$

(22) $$s.t.\;\;\;\sum_{S}\sum_{A}\phi_{i,\pi_{-i}}\left(S_{i},A_{i}\right)c\left(S_{i},A_{i}\right)\leq \theta_{E}$$

(23) $$\sum_{A}\phi_{i,\pi_{-i}}\left(S_{i}^{\prime },A_{i}\right)=\sum_{S}\sum_{A}\phi_{i,\pi_{-i}}\left(S_{i},A_{i}\right)P\left[S_{i}^{\prime }|S_{i},A_{i}\right]$$

(24) $$\sum_{S}\sum_{A}\phi_{i,\pi_{-i}}\left({S_{i}}^{\prime },A_{i}\right)=1$$

(25) $$\phi_{i,\pi_{-i}}\left({S_{i}}^{\prime },A_{i}\right)\geq 0$$

The objective function in (21) maximizes the probability that the accurate location of IoT device *i* is provided to a corresponding device. On the other hand, the constraint in (22) maintains the average energy outage probability below the desired target probability $\theta_{E}$. The constraint in (23) is for the Chapman-Kolmogorov equation. In addition, we can satisfy the probability properties with the constraints in (24) and (25).

If the LP problem is feasible, the stationary best response policy of IoT device *i* can be given by

(26) $$\pi_{i}^{*}\left(S_{i},A_{i}\right)=\frac{\phi_{i,\pi_{-i}}^{*}\left(S_{i},A_{i}\right)}{\sum_{A_{i}^{\prime }\in A_{i}}\phi_{i,\pi_{-i}}^{*}\left(S_{i},A_{i}^{\prime }\right)}.$$

**Figure 2:**
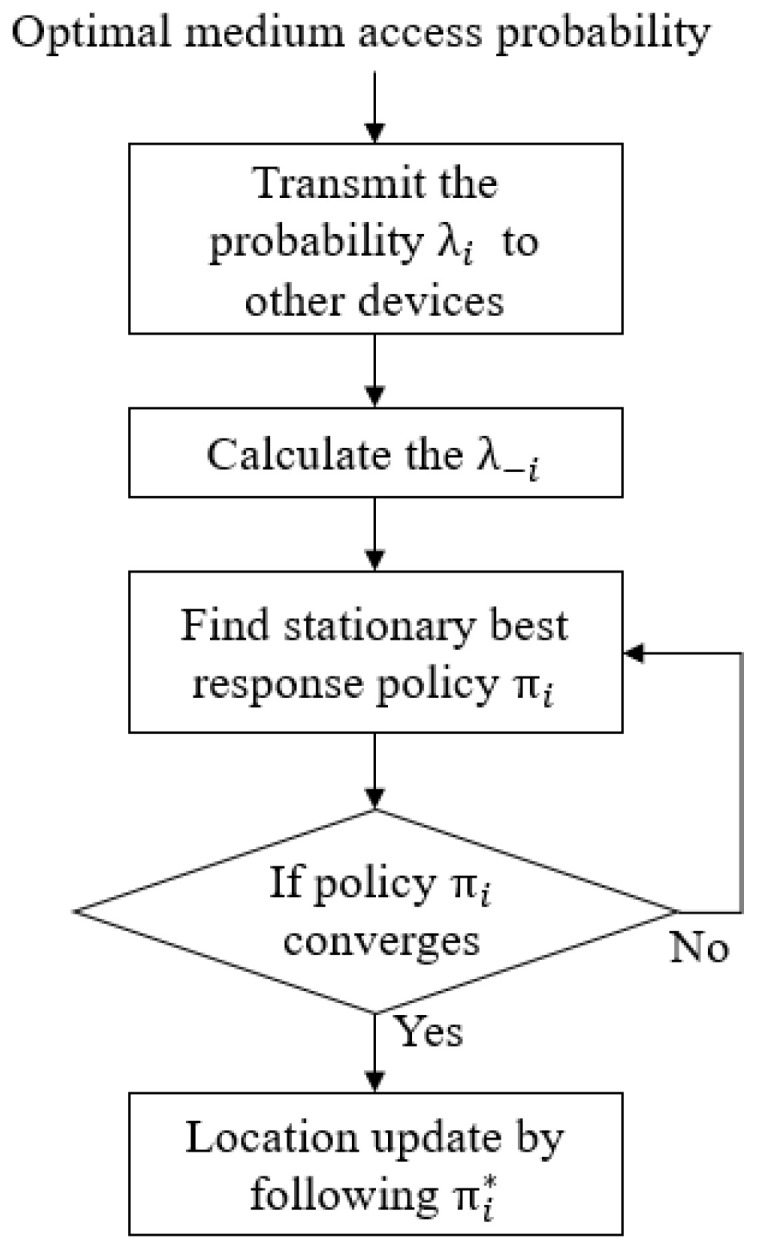
Diagram of IoT device *i* in DGLU.

The best response dynamics can be applied to update the policies of IoT devices as shown in Algorithm 1, and the diagram for IoT device *i* is shown in Figure 2. Note that the proposed algorithm is enhanced compared to the algorithm in [32] to reduce the number of time slots to exchange information (i.e., $\lambda_{i}$). In the proposed algorithm, IoT devices interact with each other through the probability $\lambda_{-i}$ (see line 6 in Algorithm 1). The probability that IoT device *i* updates its location can be obtained from

(27) $$\begin{array}{c} \lambda_{i}=\sum_{S_{i}\neq 0}\phi_{i,\pi_{-i}}\left(S_{i},A_{i}=1\right). \end{array}$$

$\lambda_{-i}$ can then be calculated by

(28) $$\begin{array}{c} \lambda_{-i}=1-\prod_{i^{\prime }\neq i}\left(1-\lambda_{i^{\prime }}\right). \end{array}$$

**Algorithm 1:** Best response dynamics-based algorithm.  1: Calculate the optimal medium access probability $P_{M}^{*}$.  2: Inform the $P_{M}^{*}$ to IoT devices  3: Initialize the policies $\pi_{i}$ for ∀i.   4: **while** Stationary policies of all IoT devices converge   5: **for** All IoT devices *i*
**do**  6: Transmit the $\lambda_{i}$ to other IoT devices with $P_{M}^{*}$  7: Calculate the probability $\lambda_{-i}=1-\prod_{i^{\prime }\neq i}\left(1-\lambda_{i^{\prime }}\right)$  8: Solve the LP problem to get the stationary best response policy $\pi_{i}^{*}$  9: **end for**   10: **end while**

To calculate $\lambda_{-i}$, each IoT device transmits the probability $\lambda_{i}$ to the other IoT devices, which implies that each IoT device should access the medium. It is assumed that IoT devices use a slotted-ALOHA protocol due to its simplicity, i.e., IoT devices access the medium at each time slot with a specific probability in a distributed manner. To reduce the collision probability, the location management server informs the optimal medium access probability to IoT devices (see line 2 in Algorithm 1). Please note that the optimal medium access probability is inversely proportional to the number of IoT devices [33].

The complexity of the whole algorithm depends on the complexity of the algorithm solving LP problem. Specifically, the complexity of the Vaidya’s algorithm, that is the representative algorithm solving a LP problem, is $O({\left(\left|S_{i}||A_{i}\right|\right)}^{3})$ [34] (i.e., polynomial time), where $\left|S_{i}\right|$ and $\left|A_{i}\right|$ denote the numbers of states and action of IoT device *i*, respectively. Meanwhile, to reach the equilibrium, the algorithm needs only a few iterations (see Section 5.1), which indicates that the proposed algorithm can easily be implemented in real IoT systems.

## 5. Evaluation Results

For a performance evaluation, we compare the proposed algorithm, DGLU, with the following three schemes: (1) ALWAYS, where an individual IoT device always performs location update whenever it moves to another location; (2) RAND, where IoT devices randomly perform location update; (3) *P*-BASED, where IoT devices perform location update with probability *P*; and (4) LEADER, where a only group leader conducts location update whenever it moves to another location. For *P*-BASED, *P* is set to 0.3.

The performance metrics are the average energy outage probability $\zeta_{E}$ and the average location accuracy $\zeta_{A}$. We assume that the initial batteries of all IoT devices are fully charged. Meanwhile, the other default parameter settings are as follows. The total number of IoT devices in the group $N_{D}$ is 3. The maximum battery capacity of the IoT devices, $E_{\max}$, is set to 10, and the number of locations within the target area $L_{\max}$ is 10. The other default parameter settings are summarized in Table 2, where [0.5 0.4 0.3] denotes the energy harvesting probability of each devices (e.g., the $P_{H}$ of IoT device 1 is 0.5).

### 5.1. Convergence to Nash Equilibrium

**Figure 3:**
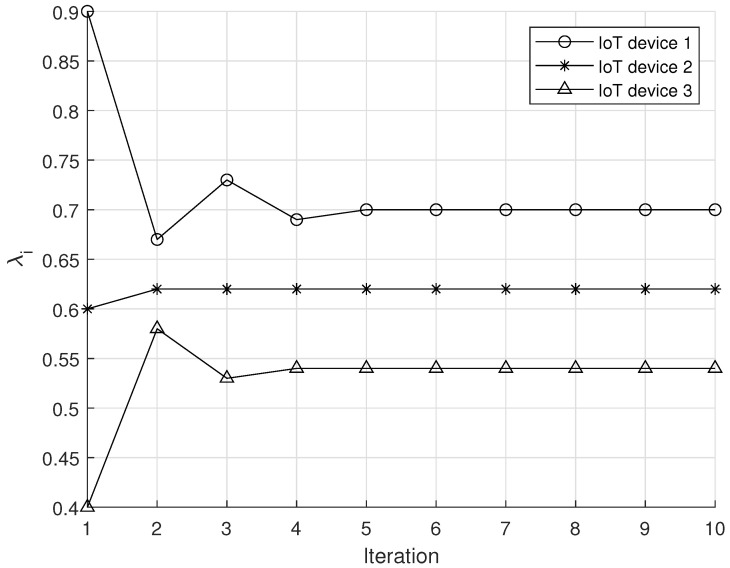
Convergence to the constrained Nash equilibrium policy.

Figure 3 shows the convergence to the constrained Nash equilibrium policy. From Figure 3, it can be observed that the proposed algorithm converges quickly within a few iterations (i.e., 6 iterations) to the equilibrium. As a result, we believe that DGLU can be implemented in practical systems without an excessively large overhead.

In addition, it can be found that when a certain IoT device performs location update with a high probability, other IoT devices conduct fewer location updates at best response. In our simulation setting, because IoT device 1 performs the location update with a high probability (i.e., 0.9) at the first iteration, other IoT devices (i.e., IoT devices 2 and 3) perform location updates with low probabilities. In this way, IoT devices 2 and 3 can reduce their energy consumption without decreasing location accuracy owing to the update by IoT device 1.

**Figure 4:**
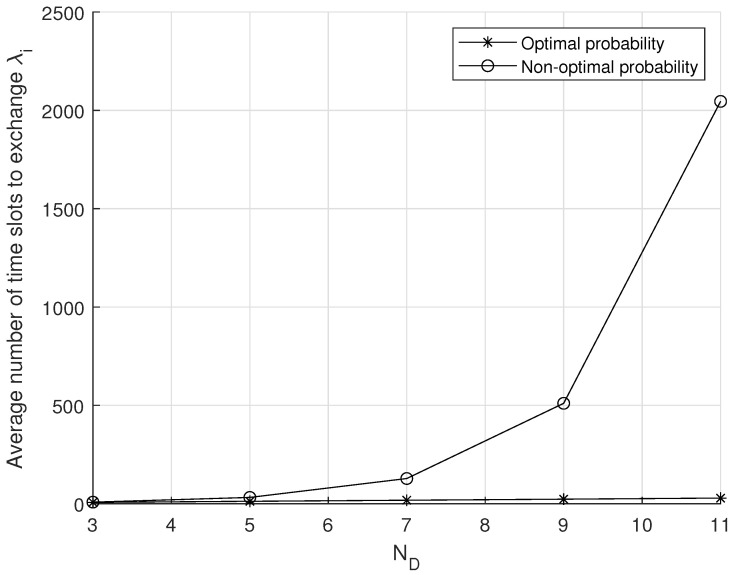
Average number of time slots to exchange $\lambda_{i}$.

Figure 4 shows the average number of time slots to exchange update probability $\lambda_{i}$ according to the number of IoT devices in a group $N_{D}$. In this result, we compare the slotted-ALOHA protocol with the optimal medium access probability to that with a non-optimal medium access probability. From Figure 4, it can be shown that the average number of time slots of the slotted-ALOHA protocol with the optimal medium access probability maintains at a low level even though $N_{D}$ increases, which indicates that IoT devices can exchange their information without high energy consumption. This is because there are few collisions due to the optimal medium access probability.

### 5.2. Effect of $\rho_{L}$

**Figure 5:**
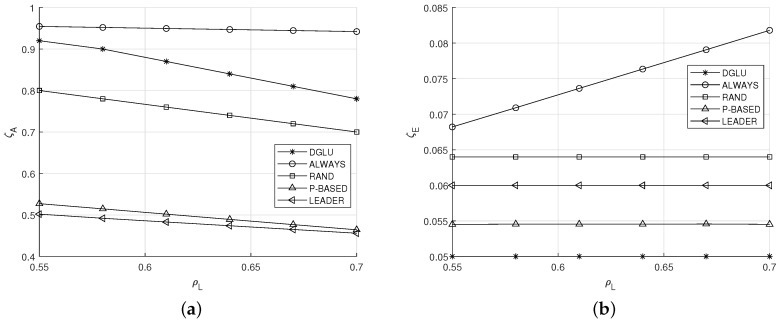
Effect of the location change probability $\rho_{L}$. (**a**) Average location accuracy $\zeta_{A}$. (**b**) Average energy outage probability $\zeta_{E}$.

Figure 5a,b shows the effect of the location change probability $\rho_{L}$ on the average location accuracy $\zeta_{A}$ and the average energy outage probability $\zeta_{E}$, respectively. A larger $\rho_{L}$ allows IoT devices to change their location more frequently. In this situation, from Figure 5a, it can be seen that the $\zeta_{A}$ of all schemes decreases. This can be explained as follows. Larger $\rho_{L}$ means that more location updates are needed to maintain the same average location accuracy $\zeta_{A}$. However, IoT devices sometimes cannot conduct location updates due to their energy depletion, which leads the reduction of the average location accuracy. However, in DGLU, IoT devices with a high energy harvesting probability update their locations more aggressively than other IoT devices with a low energy harvesting probability especially when $\rho_{L}$ is high, and thus the $\zeta_{A}$ of DGLU can be maintained at a high level (i.e., approximately 80%) without the increased energy outage probability as shown in Figure 5b. Note that, even when IoT devices with a high energy harvesting probability update their locations more aggressively, their energy is probably not depleted. Meanwhile, because ALWAYS always performs location update, its location accuracy is highest at the expense of the high energy outage probability (see Figure 5a,b).

### 5.3. Effect of $P_{h}$

**Figure 6:**
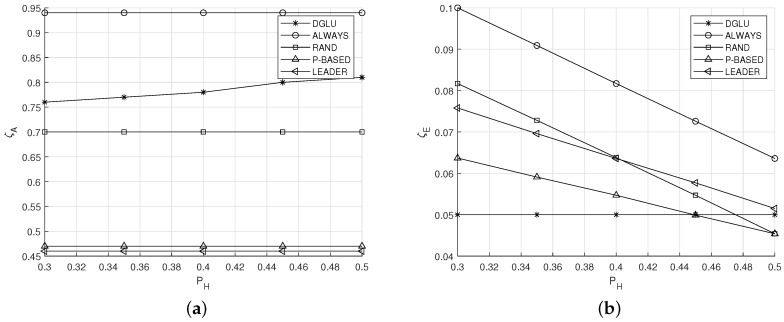
Effect of the energy harvesting probability $P_{H}$. (**a**) Average location accuracy $\zeta_{A}$. (**b**) Average energy outage probability $\zeta_{E}$.

Figure 6a,b shows the effect of the energy harvesting probability $P_{H}$ on the average location accuracy $\zeta_{A}$ and the average energy outage probability $\zeta_{E}$, respectively. From Figure 6a, it can be observed that the $\zeta_{A}$ of DGLU increases with the increase in $P_{H}$, whereas the $\zeta_{A}$ of the other schemes is constant regardless of $P_{H}$. This can be explained as follows. A large $P_{H}$ implies that the energy of IoT devices is not depleted even when they frequently conduct location update. In DGLU, IoT devices recognize this situation, and thus update their location frequently. However, the other comparison schemes follow fixed location update policies regardless of $P_{H}$. Thus, their energy outage probabilities decrease (see Figure 6b).

Meanwhile, from Figure 6, it can be found that the $\zeta_{A}$ of LEADER is lowest. This can be explained as follows. In LEADER, a group leader always updates its location. Thus, its energy can be depleted with high probability, which implies the location management server cannot maintain the accurate location information of any IoT devices.

### 5.4. Effect of $\theta_{E}$

**Figure 7:**
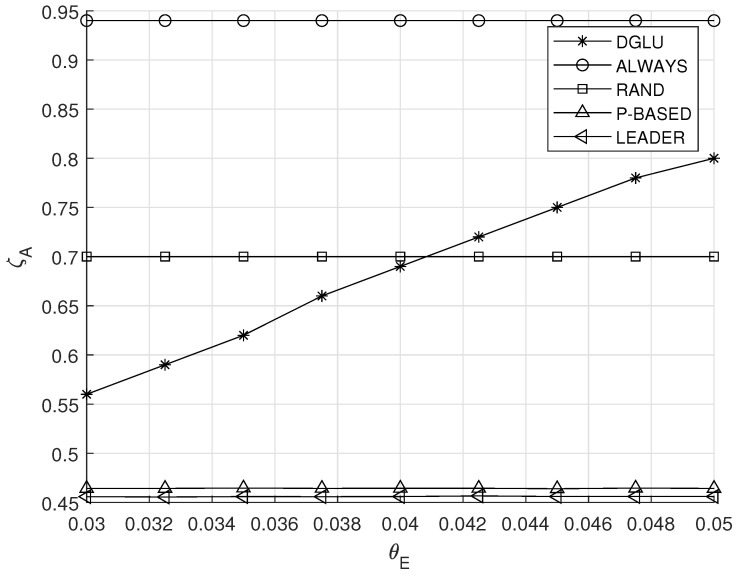
Effect of the target energy outage probability $\theta_{E}$ on the average location accuracy $\zeta_{A}$.

Figure 7 demonstrates the effect of the target energy outage probability $\theta_{E}$ on the average location accuracy $\zeta_{A}$. As $\theta_{E}$ increases, IoT devices can perform more location updates with less concern about energy depletion. By taking this situation into account, IoT devices in DGLU conduct more location updates, which explains the increased average location accuracy shown in Figure 7. On the other hand, the other comparison schemes conduct location update in accordance with the fixed policy, and therefore their average location accuracy is constant.

### 5.5. Effect of $N_{d}$

**Figure 8:**
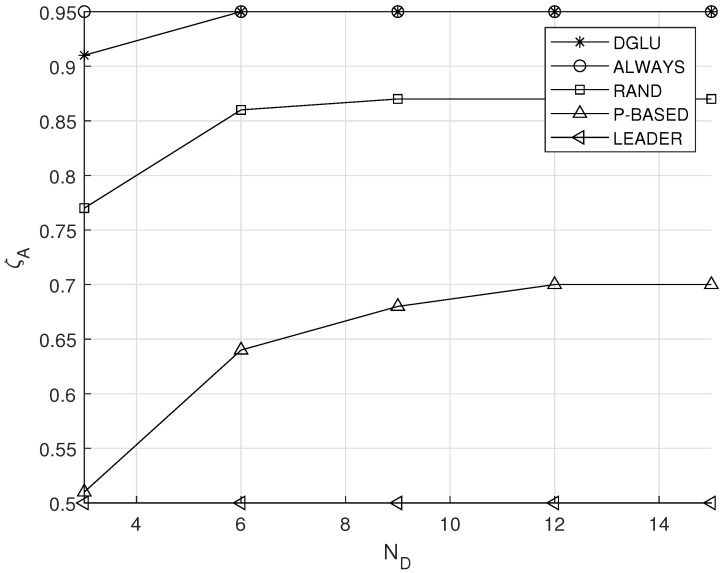
Effect of the number of IoT devices in one group $N_{D}$ on the average location accuracy $\zeta_{A}$.

Figure 8 demonstrates the effect of the number of IoT devices in one group $N_{D}$ on the average location accuracy $\zeta_{A}$. As the number of IoT devices in one group increases, $\zeta_{A}$ of all schemes except ALWAYS increases and converges to certain values. This is because large $N_{D}$ indicates low probability that all IoT devices who decide to conduct the location update cannot do that due to the energy depletion (which causes the decrease of $\zeta_{A}$). However, this probability becomes 0 when $N_{D}$ is more than a certain number (which causes the convergence of $\zeta_{A}$).

## 6. Conclusions and Future Work

In this paper, we proposed a distributed group location update algorithm (DGLU). In DGLU, geographically proximate IoT devices decide whether to update their location in a distributed manner by means of a constrained stochastic game model. We introduced a best response dynamics-based algorithm to obtain a multi-policy constrained Nash equilibrium. The evaluation results demonstrated that DGLU can achieve an accuracy of location information that is comparable with that of the individual location update scheme while guaranteeing a low energy outage probability. Moreover, it can be demonstrated that the proposed algorithm is not high, and DGLU operates adaptively even when the operating environments (i.e., the location change probability and the energy harvesting probability) change. In our future work, we will develop a method to estimate the energy harvesting probabilities of IoT devices for more sophisticated operation.

## Figures and Tables

**Table 1:** Summary of notations.

Notation	Description
$S_{i}$	Local state space of IoT device *i*
*S*	Global state space
$L_{i}$	State space for denoting the current location of IoT device *i*
$U_{i}$	State space for denoting the registered location of IoT device *i* at the location management server
$E_{i}$	State space for denoting the energy of IoT device *i*
$L_{\max}$	Number of locations within the target area
$E_{\max}$	Maximum battery capacity of IoT device
$A_{i}$	Local action space of IoT device *i*
*A*	Global action space
$\rho_{L_{i}}$	Probability of occurrence of location change of IoT device *i*
$P_{L_{i}L_{i}^{\prime }}$	Probability that IoT device *i* moves from $L_{i}$ to $L_{i}^{\prime }$
$P_{H}$	Probability that IoT device harvests one unit of energy
$\pi_{i}$	Stationary policy of mobile device *i*
π	Stationary multi-policy of all mobile devices
$\pi_{-i}$	Stationary policy of all edge clouds except mobile device *i*
$\lambda_{-i}$	Probability that at least one IoT device except IoT device *i* has registered its current location with the location management server

**Table 2:** Default Parameter Settings.

Parameter	$E_{\max}$	$\rho_{L}$	$\theta_{E}$	$P_{H}$
Value	10	0.7	0.05	[0.5 0.4 0.3]
